# Regulatory T Cells Protect Fine Particulate Matter-Induced Inflammatory Responses in Human Umbilical Vein Endothelial Cells

**DOI:** 10.1155/2014/869148

**Published:** 2014-05-29

**Authors:** Wen-cai Zhang, Yan-ge Wang, Zheng-feng Zhu, Fang-qin Wu, Yu-dong Peng, Zhu-yue Chen, Jin-hua Yang, Jing-jing Wu, Yi-tian Lian, Mei-an He, Tang-chun Wu, Long-xian Cheng

**Affiliations:** ^1^Laboratory of Cardiovascular Immunology, Institute of Cardiology, Union Hospital, Tongji Medical College of Huazhong University of Science and Technology, Wuhan 430000, China; ^2^Institute of Occupational Medicine and the Ministry of Education Key Lab of Environment and Health, Huazhong University of Science and Technology, Wuhan 430000, China; ^3^Institute of Cardiology, LIYUAN Hospital, Tongji Medical College of Huazhong University of Science and Technology, Wuhan 430000, China

## Abstract

*Objective*. To investigate the role of CD4^+^CD25^+^ T cells (Tregs) in protecting fine particulate matter (PM-) induced inflammatory responses, and its potential mechanisms. *Methods*. Human umbilical vein endothelial cells (HUVECs) were treated with graded concentrations (2, 5, 10, 20, and 40 µg/cm^2^) of suspension of fine particles for 24h. For coculture experiment, HUVECs were incubated alone, with CD4^+^CD25^−^ T cells (Teff), or with Tregs in the presence of anti-CD3 monoclonal antibodies for 48 hours, and then were stimulated with or without suspension of fine particles for 24 hours. The expression of adhesion molecules and inflammatory cytokines was examined. *Results*. Adhesion molecules, including vascular cell adhesion molecule-1 (VCAM-1) and intercellular adhesion molecule-1 (ICAM-1), and inflammatory cytokines, such as interleukin (IL-) 6 and IL-8, were increased in a concentration-dependent manner. Moreover, the adhesion of human acute monocytic leukemia cells (THP-1) to endothelial cells was increased and NF-**κ**B activity was upregulated in HUVECs after treatment with fine particles. However, after Tregs treatment, fine particles-induced inflammatory responses and NF-**κ**B activation were significantly alleviated. Transwell experiments showed that Treg-mediated suppression of HUVECs inflammatory responses impaired by fine particles required cell contact and soluble factors. *Conclusions*. Tregs could attenuate fine particles-induced inflammatory responses and NF-**κ**B activation in HUVECs.

## 1. Introduction

Particulate air pollution caused by fine particles with aerodynamic diameters under 2.5 *μ*m (PM_2.5_) is well known to be associated with the morbidity and mortality of cardiovascular diseases [1, 2]. Epidemiological studies have reported that fine particulate matter is a risk factor for the mortality of cardiovascular diseases through mechanisms that may include pulmonary and systemic inflammation, accelerated atherosclerosis, and altered cardiac autonomic functions [3]. Previous animal studies also showed that long-term exposure to low concentrations of PM_2.5_ caused significant increase in plaque areas and macrophage infiltration, likely via vascular inflammation, and increased the generation of reactive oxygen species [4, 5]. In diabetes, exposure to PM_2.5_ has been found to induce excessive reactive oxygen species and endothelial dysfunction, which may in turn enhance the risk of cardiovascular diseases [6]. However, to date, the underlying pathophysiological mechanisms connecting fine particles and cardiovascular diseases, especially atherosclerosis, remain unclear.

Inhaled insoluble PM_2.5_ and smaller PM_0.1_ have been shown to quickly translocate into the circulation from lungs, with the potential exerting direct effects on homeostasis and cardiovascular integrity [7]. As a result, the barrier functions of the endothelium may be damaged by PM_2.5_ in the circulation. Several* in vivo* experiments previously found that intratracheal instillation with particles led to systemic microvascular dysfunction [8, 9]. In addition,* in vitro* studies also suggested that particles may activate endothelial cells and induce the expression of adhesion molecules, including vascular cell adhesion molecule-1 (VCAM-1) and intercellular adhesion molecule-1 (ICAM-1), and inflammatory cytokines, such as interleukin (IL-) 6 and IL-8, in endothelial cells [10–15]. Since endothelial activation may lead to an increased risk of cardiovascular events [16], the effects of particles (SRM2786 < 4 *μ*m) used in this study on human umbilical vein endothelial cells (HUVECs) were first investigated by examining the expression of specific adhesion molecules and inflammatory cytokines.

Regulatory T (Treg) cells belong to a unique lineage of T cells that play an important role in the modulation of immune responses and the reduction of deleterious immune activation owing to their immunoregulatory and immunosuppressive functions [17]. A previous study showed that Treg cells were able to protect the proinflammatory activation in HUVECs exposed to oxidized low-density lipoprotein (ox-LDL) or lipopolysaccharide (LPS) by directly interacting with target endothelial cells and promoting the secretion of IL-10 and transforming growth factor-*β*1 [18]. However, the role of Treg cells in fine particulate matter-induced inflammatory responses and endothelial functions has not yet been elucidated. Therefore, in the present study, we further observed the effects of Treg cells on fine particles-induced inflammatory responses and endothelial functions in HUVECs and explored its potential mechanisms.

## 2. Materials and Methods

### 2.1. Ethical Statement

The investigation conforms to the principles outlined in the Declaration of Helsinki. The trial was approved by the ethics committee of Tongji Medical College of Huazhong University of Science and Technology. And all volunteers provided written informed consent to participate in the study.

### 2.2. Particle Samples

In this study, urban fine particulate matter (<4 *μ*m) (SRM2786) was obtained from the National Institute of Standards and Technology. The particles were treated by sonicating a 10000 *μ*g/mL suspension in cell culture medium for 30 min in cycles for 10 min each, after which the suspension of particles was frozen and stored at −20°C. Before each experiment, the suspension was thawed and sonicated for 15 min and then immediately diluted to the assigned concentrations in cell culture medium.

### 2.3. HUVEC Cultures

HUVECs were derived from human umbilical veins that were cannulated, washed with Hanks' solution to wipe off blood, and then digested with 1% collagenase (Sigma, USA) for 15 min at 37°C. After removal of collagenase, cells were incubated at 37°C on gelatin-coated culture dishes in Ml99 medium (Gibco, USA) and supplemented with 20% fetal calf serum (Gibco), 100 *μ*g/mL heparin (Sigma), 50 *μ*g/mL endothelial cell growth factor (Gibco), 25 mM Hepes buffer, 2 mM L-glutamine, 100 U/mL penicillin, and 100 U/mL streptomycin, as previously described [19]. Cells between passages 2 and 6 were used for experiments. The phenotype of HUVECs was verified by von Willebrand antigen staining.

### 2.4. THP-1 Cultures

The monocytic cell line THP-1 was obtained from the American Type Culture Collection (Manassas, USA) and cultured in RPMI1640 with 10% fetal calf serum.

### 2.5. Isolation and Purification of Tregs

Peripheral blood was collected from 20 normal volunteers, and peripheral blood mononuclear cells (PBMCs) were isolated using Ficoll-Paque PLUS (GE Healthcare, USA). Treg cells were subsequently isolated using the Human CD4^+^CD25^+^ Regulatory T Cell Isolation Kit (Miltenyi Biotec, Germany) according to the manufacturer's instructions. In brief, PBMCs were labeled with a mixture of biotin-conjugated antibodies and anti-biotin microbeads, and CD4^+^ cells were then obtained by negative selection. Next, CD4^+^CD25^+^ Treg cells were isolated twice by positive selection to achieve higher purity. The purity of the CD4^+^CD25^+^ cell population was >90% as assessed by FACS.

### 2.6. Functional Suppression Assays

CD4^+^CD25^−^ T cells (Teff) and CD4^+^CD25^+^ T cells (Tregs) were cocultured in 96-well plates coated with 50 ng/mL anti-CD3 mAb (eBioscience, USA) at a density of 10^4^ cells/well with different Teff/Treg ratios (1 : 1, 1 : 1/2, 1 : 1/4, and 1 : 1/8). All wells were cultured in a final volume of 200 *μ*L with the presence of T cell-depleted and irradiated antigen presenting cells (10^5^ cells/well). After 72 h, [3H]-thymidine (1 *μ*Ci/well) was added for 16 h prior to the determination of proliferation by scintillation counting (MicroBeta1450 Liquid Scintillation Counter; Perkin Elmer, USA). Percent inhibition of proliferation was determined as follows: (1-[3H]-thymidine uptake of cocultured Treg and Teff)/Teff alone × 100%. Triplicate wells were used in all suppression experiments.

### 2.7. Cells Stimulations

Confluent HUVECs were growth arrested by serum deprivation for 24 h. In order to explore the optimum concentration of the particles to stimulate HUVECs, cells were treated with graded concentration (2, 5, 10, 20, and 40 *μ*g/cm^2^) of suspension of the particles for 24 h. In some experiment, cells were pretreated for 30 min with the NF-*κ*B inhibitor PDTC (10 *μ*mol/L) (Sigma, USA) before stimulation with PM (20 *μ*g/cm^2^) for 24 h. Sometimes, LPS (1 *μ*g/mL) was selected as a positive control. Then, the cells were harvested and supernatant was collected for further assay.

### 2.8. Coculture of HUVECs and Tregs

For synchronization, HUVECs were cultured in 6-well plates containing serum-free medium for 24 h when the cells were grown to 80–90% confluence. Nonadherent cells were washed off with PBS, and new culture medium was replaced. Next, HUVECs and T cells (2 : 1) were cocultured as previously described [20]. Briefly, HUECVs (1 × 10^6^/well) were incubated alone or with CD4^+^CD25^−^ or CD4^+^CD25^+^ T cells for 48 h in the presence of 50 ng/mL anti-CD3 mAb, followed by addition of PM (20 *μ*g/cm^2^) or LPS (1 *μ*g/mL) for another 24 h. After incubation, floating T cells were discarded, and HUVECs were washed with PBS and harvested. Finally, supernatants were collected and kept frozen at −80°C for further experiments.

### 2.9. Flow Cytometry for Detection of VCAM-1

After the coculture period, HUVECs were digested with 0.25% trypsin without EDTA and washed two times with PBS. Cells were then stained with PE-anti-human VCAM-1 antibody (eBioscience, USA) for 30 min at 4°C. Isotype control antibodies were used to ensure antibody specificity. Stained cells were detected by a FACSAria flow cytometer (BD Biosciences, USA), and the percentage of positive cells was analyzed by FlowJo 7.6.1.

### 2.10. Enzyme-Linked Immunosorbent Assay

Supernatants derived from different groups were subjected to specific ELISA assays (all from R&D Systems, USA) according to the manufacturer's instructions. The minimum detectable concentrations for sVCAM-1, sICAM-1, IL-6, IL-8, TGF-*β*1, and IL-10 were 1.26 ng/mL, 0.254 ng/mL, 0.7 pg/mL, 7.5 pg/mL, 15.4 pg/mL, and 3.9 pg/mL, respectively. The intra-assay and interassay coefficients of variation for these ELISA assays were <5% and <10%, respectively. All measurements were taken twice.

### 2.11. Real-Time PCR

Total RNA of HUVECs from different groups was extracted using Trizol Reagent (Takara, Japan) according to the manufacturer's instruction and then subjected to cDNA synthesis using the RNA PCR Kit (Takara). The mRNA expression was determined with the use of SYBR Green Master Mix (Takara) on an ABI Prism 7900 sequence detection system (Applied Biosystems, USA). For each sample, the mRNA expression was normalized to *β*-actin. Primers used in this study were shown in Table 1.

### 2.12. Adhesion of THP-1 Cells to Endothelial Cells

After the coculture period, THP-1 cells were labeled with CFSE (Sigma, USA) according to the manufacturer's instructions and added to endothelial cell monolayers grown in 24-well plates at a monocyte-to-endothelial cell ratio of 10 : 1. After a 1 h culture at 37°C, suspension cells were removed by three washes with PBS. Subsequently, cells were fixed with 4% paraformaldehyde, and the number of green fluorescent adherent cells was counted in five randomly chosen fields under a fluorescence microscope.

### 2.13. Transwell Experiment

Transwell experiments were conducted in 24-well plates (0.4 *μ*m pore size, Corning Costar, USA) by culturing HUVECs (1 × 10^6^/mL) in the lower well and the Treg cells (5 × 10^5^/mL) with anti-CD3 mAb in the inserts. After 48 h of culture, the inserts were removed, and the HUVECs in the lower well were stimulated with PM (20 *μ*g/cm^2^) for 24 h. For neutralization experiments, neutralizing antibodies against IL-10 (5 *μ*g/mL), TGF-*β*1 (5 *μ*g/mL), or isotype control (5 *μ*g/mL) (all from R&D Systems, USA) were added at the start of the coculture in the lower wells. After the incubation period, HUVECs and supernatants were collected for further experiments.

### 2.14. Electrophoretic Mobility Shift Assay (EMSA) for Detection of NF-*κ*B

For the EMSA assay, nuclear proteins were extracted from different groups using the Nuclear and Cytoplasmic Protein Extraction Kit (Beyotime Institute of Biotechnology, China). DNA-protein interactions were detected using the LightShift Chemiluminescent EMSA Kit (Pierce, USA) according to the manufacturer's instructions. The consensus sequences of biotin-labeled NF-*κ*B oligonucleotides were as follows: forward, 5′-AGTTGAGGGGACTTTCCCAGGC-3′, and reverse, 5′-GCCTGGGAAAGTCCCCTCAACT-3′. Biotin end-labeled DNA was detected by chemiluminescence. To verify whether detected shifted bands were specific for NF-*κ*B, competition tests were conducted with the use of a 200-fold excess of unlabeled “cold” oligonucleotides, in addition to labeled probes.

### 2.15. Statistical Analysis

Data are shown as means ± SEM. Differences were evaluated using one-way ANOVA for multiple comparisons, followed by the post hoc Student-Newman-Keuls test when necessary. All analyses were done using SPSS 16.0, and statistical significance was set at *P* < 0.05.

## 3. Results

### 3.1. Identification of CD4^+^CD25^+^ T Cells

**Figure 1:**
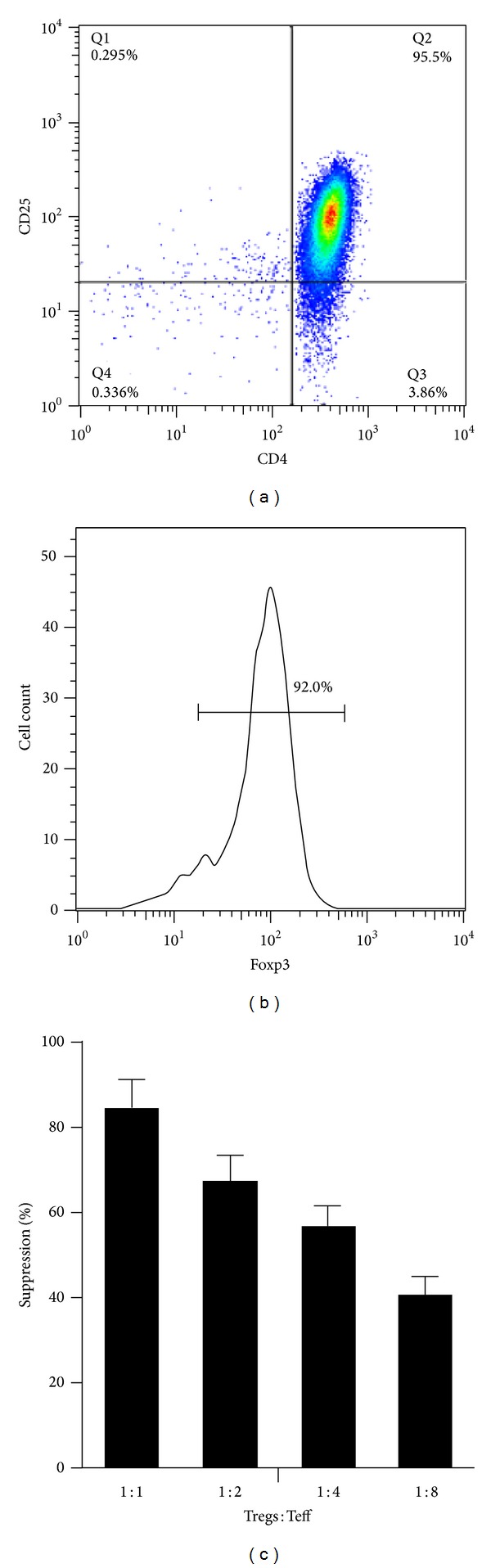
Isolation and identification of CD4^+^CD25^+^ T cells. (a) The purity of CD4^+^CD25^+^ T cells isolated from peripheral blood mononuclear cells (PBMCs) of healthy volunteers was examined by flow cytometry. (b) The percentage of the foxp3^+^ population among the sorted CD4^+^CD25^+^ T cells. (c) Proliferation was evaluated by thymidine incorporation. The relative effect of CD4^+^CD25^+^ T cells was expressed as percentage inhibition of CD4^+^CD25^−^ T cells. Experiments were repeated 3 times.

By flow cytometry, the purity of CD4^+^CD25^+^ Tregs isolated from peripheral blood mononuclear cells was found to be >90% (Figure 1(a)), and most of the isolated Tregs were Foxp3^+^ (Figure 1(b)). To test whether the cells with the phenotype of CD4^+^CD25^+^ T cells had functional characteristics of Tregs, we cocultured them with CD4^+^CD25^−^ T cells at different ratios and assessed their capacity to suppress the proliferation of autologous CD4^+^CD25^−^ T cells after activation with anti-CD3 mAb. As expected, Tregs were able to efficiently suppress the proliferation of CD4^+^CD25^−^ T cells in a dose-dependent manner (Figure 1(c)).

### 3.2. PM Induces HUVECs Inflammatory Responses in a Concentration-Dependent Manner

**Figure 2:**
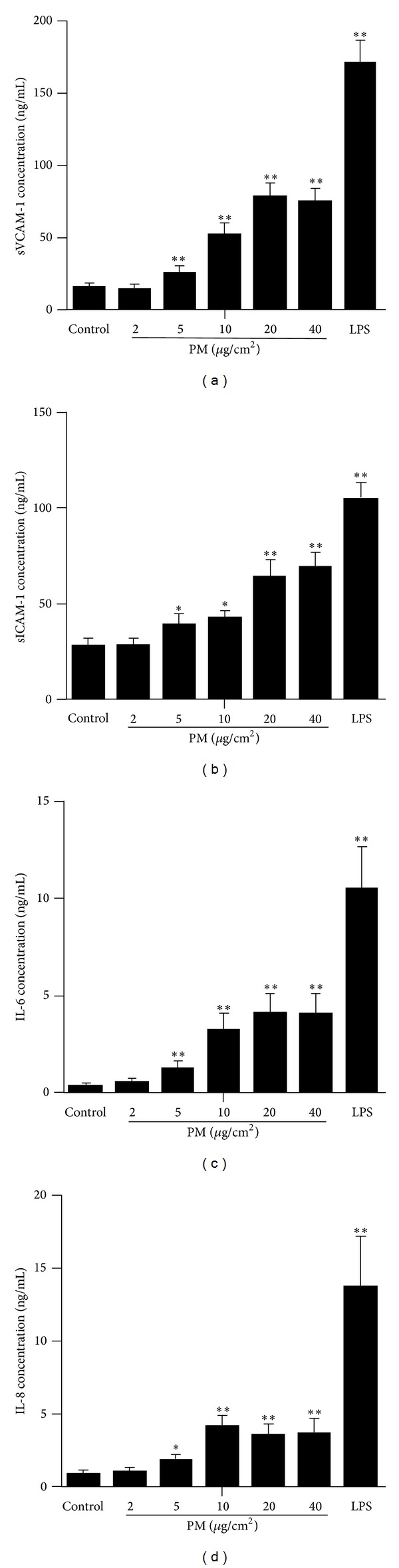
PM induces HUVECs inflammatory responses in a concentration-dependent manner. HUVECs were treated with graded concentration (2, 5, 10, 20, and 40 *μ*g/cm^2^) of suspension of the particles for 24 h and the supernatant was collected. The concentration of sVCAM-1 (a), sICAM-1 (b), IL-6 (c), and IL-8 (d) was detected by Elisa. * indicates PM or LPS versus control. *P < 0.05; **P < 0.01. Experiments were repeated 3 times.

It has been reported that PM from different sources causes adhesion molecules and cytokines expression in ECs [10–15]. However, the effect of the particles used in this study in HUVECs was not determined before. Therefore, in this study, we first investigated the effects of the particles on HUVECs by examining the expression of specific adhesion molecules (VCAM-1 and ICAM-1) and inflammatory cytokines (IL-6 and IL-8). We examined PM-induced HUVECs adhesion molecules and inflammatory cytokines expression after 24 h of stimulation with 2, 5, 10, 20, and 40 *μ*g/cm^2^. We found that particles induced inflammatory responses in a concentration-dependent manner beginning at 5 *μ*g/cm^2^ (Figure 2). The optimum concentration of PM-induced HUVECs VCAM-1, ICAM-1, IL-6, and IL-8 expression was 20, 40, 20, and 10 *μ*g/cm^2^, respectively (Figure 2). Thus, we used the concentration of 20 *μ*g/cm^2^ to stimulate cells for further experiment.

### 3.3. Tregs Alleviate VCAM-1 Expression in PM-Exposed HUVECs

**Figure 3:**
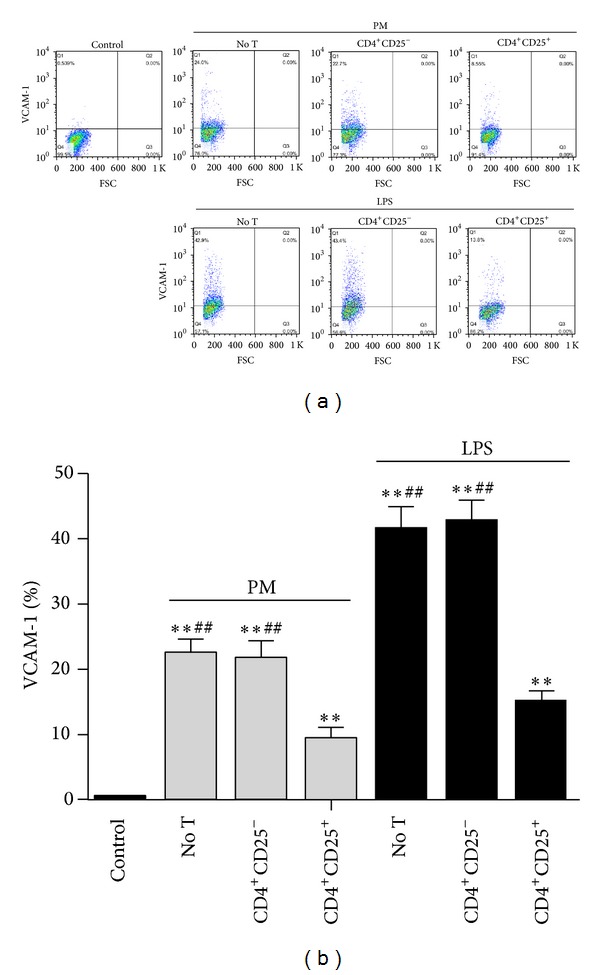
Tregs alleviate the expression of VCAM-1 in PM-exposed HUVECs. After the coculture period, HUVECs from different groups were harvested, and the VCAM-1 expression was detected by flow cytometry. (a) Dot plots showing the percentages of VCAM-1 expression in HUVECs. (b) The VCAM-1 expression in different groups of HUVECs. Data are expressed as means ± SEM. * indicates no T, CD4^+^CD25^−^, or CD4^+^CD25^+^ versus control; ^#^ indicates no T or CD4^+^CD25^−^ versus CD4^+^CD25^+^; **P < 0.01; ^##^ *P* < 0.01. Experiments were repeated 6 times.

HUVECs were culture alone or cocultured with CD4^+^CD25^−^ T cells (Teff) or Tregs in the presence of anti-CD3 mAb for 48 h and then treated with or without (control) PM/LPS for another 24 h. After the coculture time, the VCAM-1 expression in HUVECs exposed to PM was detected by flow cytometry. The results show that the VCAM-1 expression was significantly upregulated after 24 h of PM exposure in the absence of T cells, compared to the control (22.4% ± 1.9% versus 0.42% ± 0.12%; *P* < 0.01; Figures 3(a) and 3(b)). What is more, we found that Tregs-treated HUVECs showed dramatically reduced VCAM-1 expression (PM, 9.3% ± 1.5%; LPS, 14.9% ± 1.8%), compared to the group without T cells (PM, 22.4% ± 1.9%; LPS, 41.4% ± 3.5%; *P* < 0.01) or the coculture group with CD4^+^CD25^−^ T cells (PM, 21.7% ± 2.4%; LPS, 42.6% ± 3.3%; *P* < 0.01) (Figures 3(a) and 3(b)).

### 3.4. Tregs Downregulate Adhesion Molecules and Inflammatory Cytokines in PM-Exposed HUVECs

**Figure 4:**
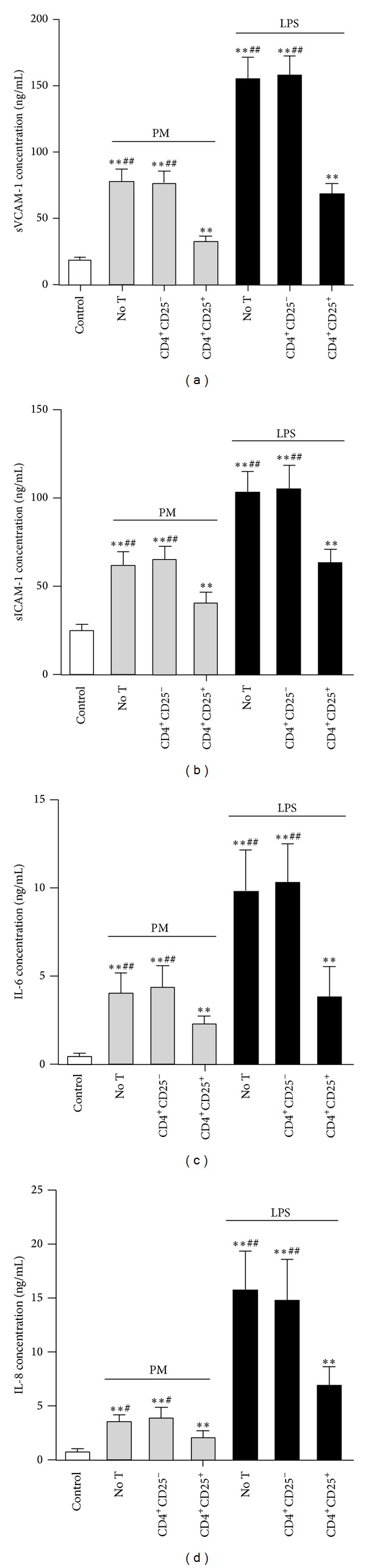
Tregs downregulate the protein expression of adhesion molecules and inflammatory cytokines in PM-exposed HUVECs. The ELISA assay was used to detect the concentration of sVCAM-1 (a), sICAM-1 (b), IL-6 (c), and IL-8 (d) in the supernatants from different groups of HUVECs. Data are expressed as means ± SEM. * indicates no T, CD4^+^CD25^−^, or CD4^+^CD25^+^ versus control; ^#^ indicates no T or CD4^+^CD25^−^ versus CD4^+^CD25^+^. *P < 0.05, **P < 0.01, ^#^ *P* < 0.05, and ^##^ *P* < 0.01. Experiments were repeated 5 times.

After the coculture period, the ELISA assay was used to detect the concentration of adhesion molecules and inflammatory cytokines. The results show that the suspension of fine particles and LPS significantly increased the protein levels of sVCAM-1 (PM, 77.2 ± 9.5 ng/mL; LPS, 154.7 ± 16.2 ng/mL), sICAM-1 (PM, 61.4 ± 7.9 ng/mL; LPS, 102.5 ± 12.1 ng/mL), IL-6 (PM, 4.0 ± 1.2 ng/mL; LPS, 9.8 ± 2.5 ng/mL), and IL-8 (PM, 3.4 ± 0.7 ng/mL; LPS, 15.7 ± 3.7 ng/mL), compared to the control (18.4 ± 2.7 ng/mL, 24.7 ± 3.2 ng/mL, 5.1 ± 1.1 ng/mL, 0.45 ± 0.21 ng/mL, and 0.84 ± 0.29 ng/mL, resp.) (all *P* < 0.01; Figure 4). In addition, compared to the group without T cells, Tregs-treated HUVECs exhibited markedly decreased concentrations of all adhesion molecules and inflammatory cytokines (*P* < 0.05), whereas CD4^+^CD25^−^ T cells had no effect (*P* > 0.05; Figure 4).

**Figure 5:**
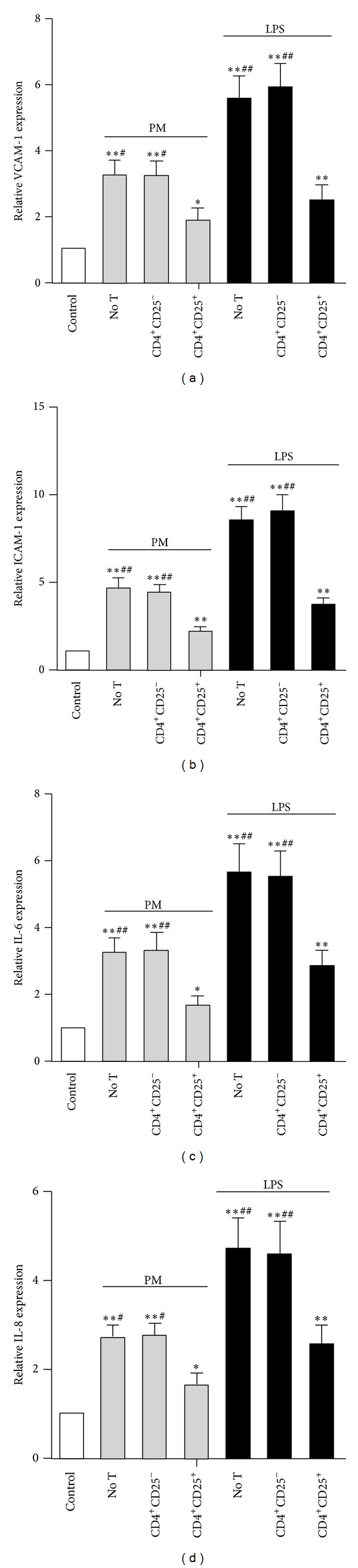
Tregs downregulate the mRNA expression of adhesion molecules and inflammatory cytokines in PM-exposed HUVECs. RT-PCR was used to detect the mRNA expression of VCAM-1 (a), ICAM-1 (b), IL-6 (c), and IL-8 (d) in different groups of HUVECs. Data are expressed as means ± SEM. * indicates no T, CD4^+^CD25^−^, or CD4^+^CD25^+^ versus control; ^#^ indicates no T or CD4^+^CD25^−^ versus CD4^+^CD25^+^. *P < 0.05, **P < 0.01, ^#^ *P* < 0.05, and ^##^ *P* < 0.01. Experiments were repeated 5 times.

The mRNA levels of VCAM-1, ICAM-1, IL-6, and IL-8 were also determined by real-time PCR (RT-PCR), and the results were normalized to the endogenous control gene *β*-actin. Consistent with the protein expression results described above, the mRNA levels of VCAM-1, ICAM-1, IL-6, and IL-8 were all increased upon PM/LPS stimulation, and Tregs were able to dramatically reduce these upregulated mRNA levels (all *P* < 0.05; Figure 5).

### 3.5. Tregs Decrease the Adhesion of THP-1 Cells to Endothelial Cells

**Figure 6:**
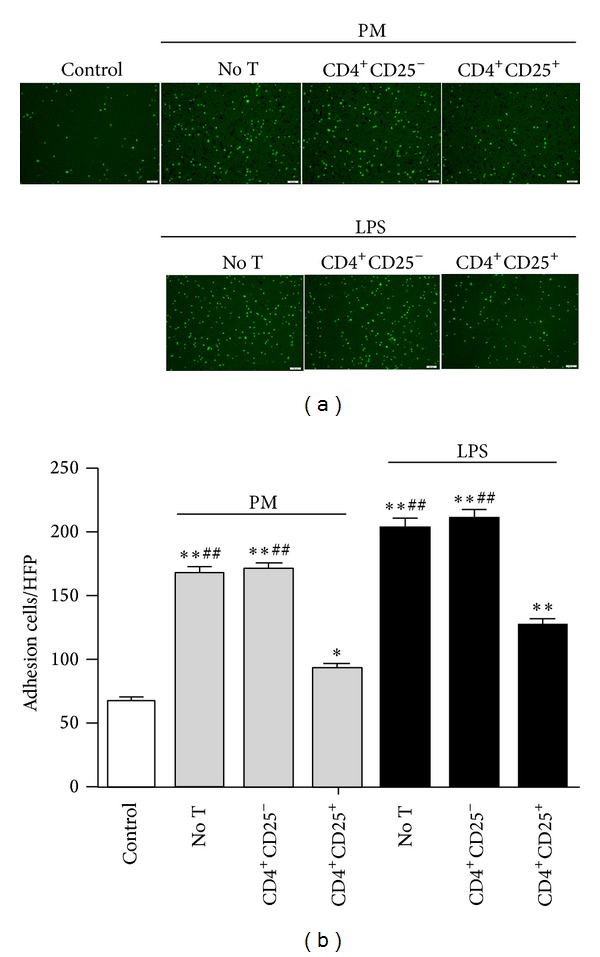
Tregs decrease the adhesion of THP-1 cells to endothelial cells. (a) Representative photomicrograph of THP-1 cells adhering to endothelial cells (ECs). THP-1 cells were identified by green fluorescence. Scale  bar = 100 nm. (b) The adhesion of THP-1 cells to ECs was determined by fluorescence microscopy. Data are expressed as means ± SEM. * indicates no T, CD4^+^CD25^−^, or CD4^+^CD25^+^ versus control; ^#^ indicates no T or CD4^+^CD25^−^ versus CD4^+^CD25^+^. *P < 0.05, **P < 0.01, and ^##^ *P* < 0.01. Experiments were repeated 4 times.

To further confirm the increases in the expression of adhesion molecules VCAM-1 and ICAM-1 in HUVECs after PM exposure, the adhesion of THP-1 cells to endothelial cells was evaluated. After the coculture period, the THP-1 cells labeled with CFSE were added to the HUVECs. As expected, the adhesion of THP-1 cells to endothelial cells was significantly increased (PM, 168 ± 5.6; LPS, 204 ± 6.9), compared to the control (67 ± 3.5) (*P* < 0.01; Figures 6(a) and 6(b)). In contrast, the adhesion of THP-1 cells to Treg-treated HUVECs was obviously reduced (PM, 93 ± 3.8; LPS, 127 ± 4.5) (*P* < 0.01), while CD4^+^CD25^−^ T cells only had a minor effect (PM, 171 ± 5.4; LPS, 211 ± 7.2) (*P* > 0.05; Figures 6(a) and 6(b)).

### 3.6. PDTC Inhibits PM-Induced Inflammatory Responses

**Figure 7:**
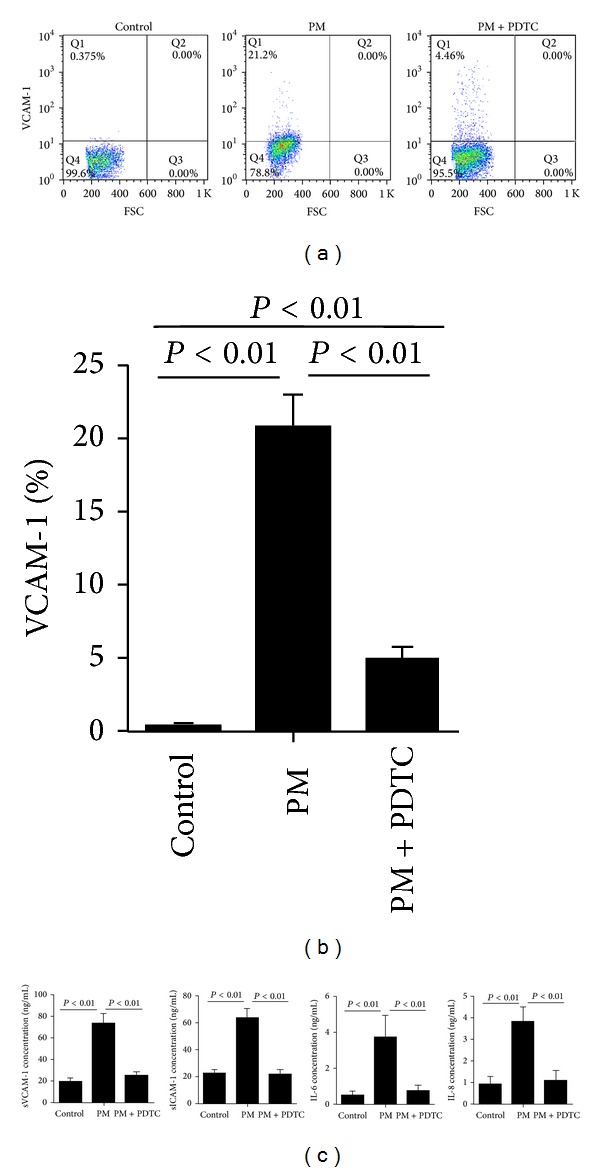
PDTC inhibits PM-induced inflammatory responses. HUVECs were pretreated for 30 min with the NF-*κ*B inhibitor PDTC (10 *μ*mol/L) before stimulation with PM (100 *μ*g/mL) for 24 h. The adhesion molecules and inflammatory cytokines were detected by flow cytometry and Elisa. (a) Dot plots showing the percentages of VCAM-1 expression in HUVECs. (b) The VCAM-1 expression in different groups of HUVECs. (c) The concentration of sVCAM-1, sICAM-1, IL-6, and IL-8 in the supernatants from different groups of HUVECs. Data are expressed as means ± SEM of 5 independent experiments.

NF-*κ*B is a transcription factor that regulates the expression of proinflammatory and antiapoptotic genes and also plays an important role in driving the inflammatory responses [21]. To test whether NF-*κ*B was involved in PM-induced inflammatory responses, we used the NF-*κ*B specific inhibitor PDTC to treat cells before PM stimulation. Form Figure 7, we demonstrated that PM-stimulated inflammatory responses were almost completely inhibited after PTDC treatment, indicating that NF-*κ*B activity might play an important role in PM-mediated inflammatory responses.

### 3.7. Tregs Downregulate PM-Induced NF-*κ*B Activation in HUVECs

**Figure 8:**
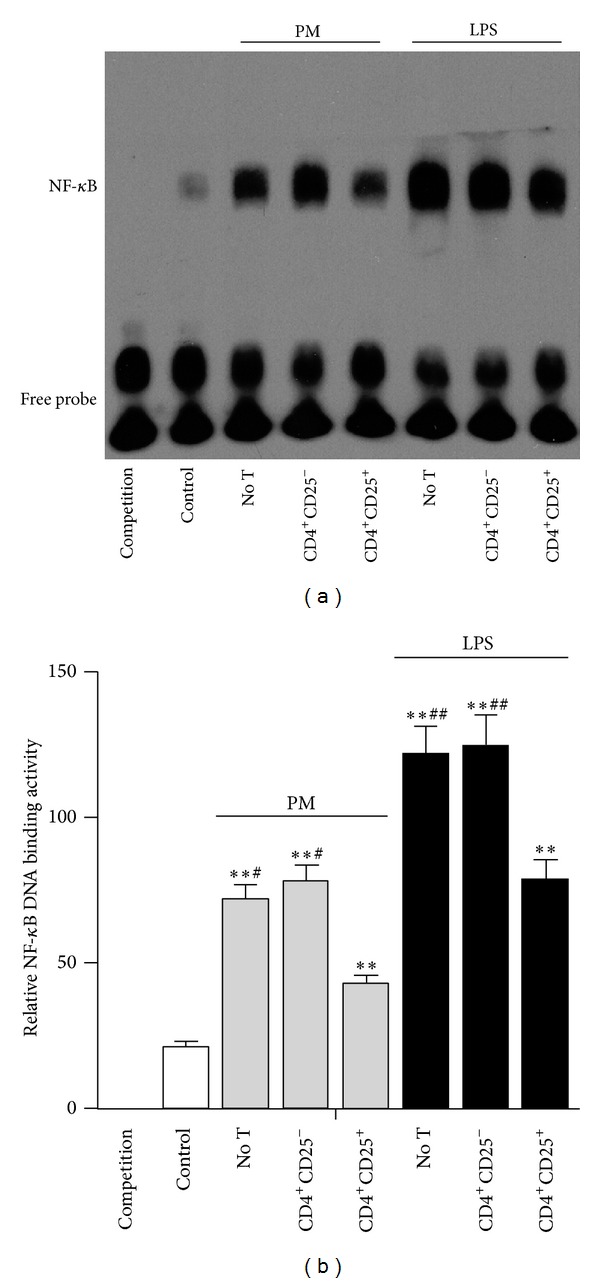
Tregs downregulate NF-*κ*B activation in HUVECs impaired by PM. The electrophoretic mobility shift assay (EMSA) was conducted with nuclear proteins isolated from different HUVEC cultures to detect the NF-*κ*B activity. (a) Representative EMSA results. (b) The DNA-binding activity of NF-*κ*B in different groups determined by the relative measurement method. Data are expressed as means ± SEM. * indicates no T, CD4^+^CD25^−^, or CD4^+^CD25^+^ versus control; ^#^ indicates no T or CD4^+^CD25^−^ versus CD4^+^CD25^+^. **P < 0.01, ^#^ *P* < 0.05, and ^##^ *P* < 0.01. Experiments were repeated 4 times.

In our study, the NF-*κ*B activity in HUVECs after PM/LPS treatment was determined by the EMSA assay using biotin-labeled oligonucleotide probes specific for the NF-*κ*B-binding sites. In agreement with the above results including upregulated levels of adhesion molecules and inflammatory cytokines, the NF-*κ*B activity was increased in HUVECs without T cells after PM or LPS stimulation, compared to the control (*P* < 0.01; Figure 8). In contrast, the decreased inflammatory responses were reflected at the transcriptional level by an obviously reduced NF-*κ*B upregulation on PM/LPS stimulation from Tregs-treated HUVECs (*P* < 0.01), whereas no difference was observed in Teff-treated HUVECs (*P* > 0.05; Figure 8).

### 3.8. Treg-Mediated Suppression of HUVECs Inflammatory Responses Is Mediated by Cell Contact and Soluble Factors

**Figure 9:**
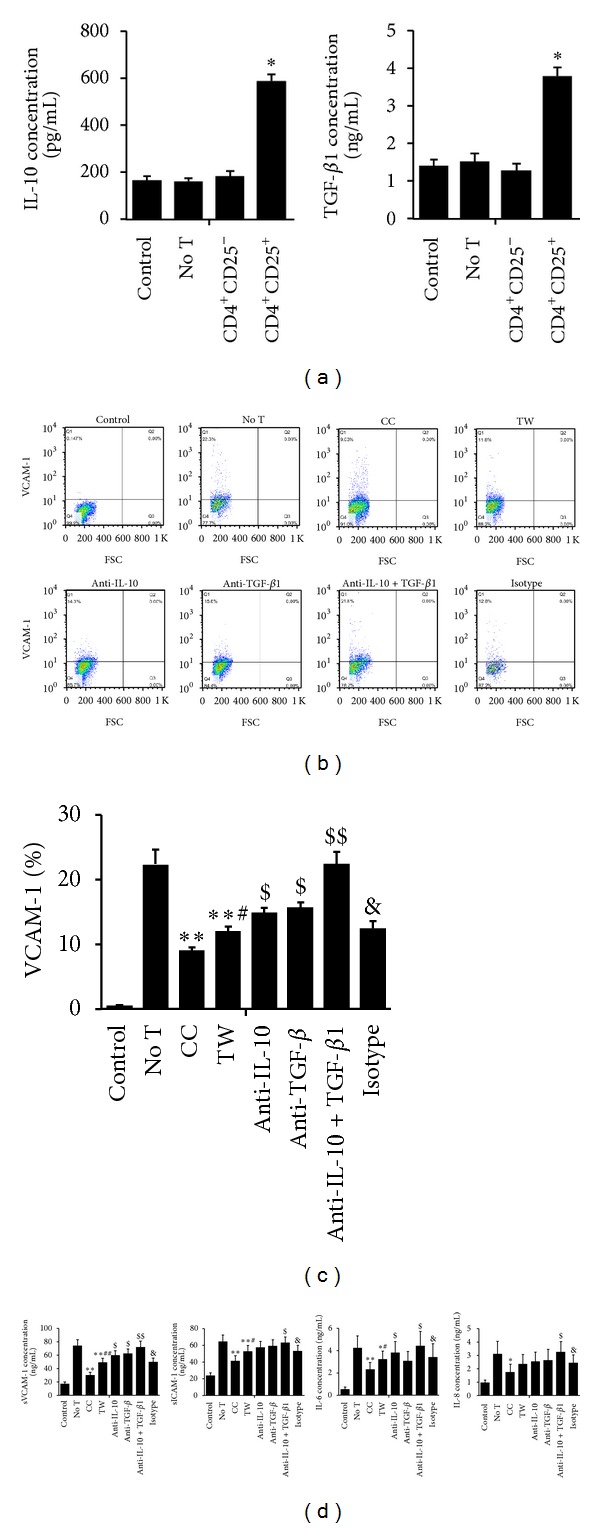
The mechanisms of Tregs-mediated suppression of HUVECs exposed to PM. HUVECs were cultured without T cells (no T) or with Tregs in the presence of anti-CD3 mAbs in either a coculture (CC) or a TW system. After 48 hours of culture, the inserts were removed and HUVECs in the lower well were stimulated with PM. In some experiments, IL-10, TGF-*β*1, IL-10 + TGF-*β*1, or isotype mAbs was added to the lower well. The adhesion molecules and cytokines were detected by flow cytometry and Elisa. (a) The concentrations of IL-10 and TGF-*β*1 in the supernatants from different groups. Data are expressed as means ± SEM of 3 independent experiments. *P < 0.01. (b) Dot plots showing the percentages of VCAM-1 expression in HUVECs. (c) The VCAM-1 expression in different groups of HUVECs. (d) The concentration of sVCAM-1, sICAM-1, IL-6, and IL-8 in the supernatants from different groups of HUVECs. Data are expressed as means ± SEM of 4 independent experiments. * indicates CC or TW versus no T; ^#^ indicates TW versus CC; ^$^ indicates versus TW; ^&^ indicates isotype versus TW. *P < 0.05, **P < 0.01, ^#^ *P* < 0.05, ^##^ *P* < 0.01, ^$^ *P* < 0.05, ^$$^ *P* < 0.01, and ^&^ *P* > 0.05.

To explore whether suppression of inflammatory responses of HUVECs exposed to PM depended on cell contact or soluble factors, we cultured HUVECs without T cells, with Treg cells in the presence of anti-CD3 mAbs in either a coculture or a TW system. After 48 hours of culture, the top compartments were removed, and the HUVECs in the lower well were treated with PM for 24 hours. By blocking physical contact between HUVECs and Tregs (TW), the suppression of adhesion molecules (VCAM-1 and ICAM-1) and inflammatory cytokines (IL-6 and IL-8) production was obviously decreased compared with coculture system (Figures 9(b), 9(c), and 9(d)). This partial reversal of suppression could be owing to the requirement of cell contact between Tregs and PM-exposed HUVECs.

It is reported that activated Tregs could produce anti-inflammatory cytokines, such as IL-10 and TGF-*β*1 [22]. What is more, we also found that the concentrations of IL-10 and TGF-*β*1 in the Tregs system was higher than that in other systems (*P* < 0.01; Figure 9(a)). To investigate whether IL-10 or TGF-*β*1 could be involved in the suppression of Tregs, the neutralizing experiments were conducted. Anti-IL-10, anti-TGF-*β*1, or isotype mAbs was added to the lower well of TW system. After treatment with anti-IL-10 mAbs or anti-TGF-*β*1, the inhibitory effects were significantly decreased; furthermore, the suppression of inflammatory responses in HUVECs was completely abolished when both anti-IL-10 and anti-TGF-*β*1 mAbs were added, while the isotype mAbs had no effect (Figures 9(b), 9(c), and 9(d)).

## 4. Discussion

Abundant epidemiological evidence indicates that PM, particularly PM_2.5_, is a major risk factor with serious consequences on the cardiovascular system [3, 23–26]. Because of its small size, PM_2.5_ could be inhaled into the lungs and translocate into the circulation, with potential direct effects on endothelial cells that lie in the innermost of blood vessels. In the present study, HUVECs were used to explore the effects of fine particles on endothelial inflammatory responses, and, for intervention studies, Treg cells isolated from healthy volunteers were employed. Consistent with previous studies, our results show that fine particles not only induced the expression of adhesion molecules and inflammatory cytokines in a concentration-dependent manner in HUVECs but also increased the adhesion of THP-1 cells to endothelial cells mainly via NF-*κ*B activation. Importantly, Treg cells were able to protect fine particles-induced inflammatory responses and downregulate NF-*κ*B activation in HUVECs via cell contact with PM-impaired HUVECs and soluble factors (mainly IL-10 and TGF-*β*1).

The endothelial barrier functions play an important role in regulating the vascular tone, cell adhesion, and vessel wall inflammation [27]. The expression levels of ICAM-1 and VCAM-1 on the membrane of endothelial cells are important markers of the activation of the endothelium [28]. These cell adhesion molecules mediate the binding of leukocytes to ECs and thereby the recruitment of leukocytes to the interstitium of the tissue [29]. The recruitment of inflammatory cells is considered the first step towards the development of atherosclerosis. Previously, PM_2.5_ and PM_10_ have been reported to induce the expression of ICAM-1 and VCAM-1 in endothelial cells [10, 12, 13]. In our study, urban fine particulate matter (<4 *μ*m; SRM2786) instead of PM_2.5_ was used to stimulate HUVECs. We found that the fine particles obviously induced both mRNA and protein expression of VCAM-1 and ICAM-1 in HUVECs, which may contribute to PM-accelerated atherosclerosis. Some animal experiments suggested that an increase in Treg cell numbers and functions is related to the reduction of atherosclerotic plaques [30–35]. In addition, Tregs have also been found to protect ox-LDL/LPS-induced expression of VCAM-1 in HUVECs [18]. Consistent with previous studies, our results show that Treg cells, but not Teff cells, significantly decreased PM-induced expression of adhesion molecules (VCAM-1 and ICAM-1) in the HUVECs.

Next, to determine whether fine particles induce the expression of adhesion molecules after 24 h of treatment, the adhesion of THP-1 cells to endothelial cells was examined. We found that compared to the control, the adhesion of THP-1 cells to PM-treated HUVECs was obviously increased, consistent with previously reported results [10, 12]. In contrast, coculture with Treg cells was able to reduce the adhesion, whereas Teff cells only had a minor effect. The adhesion of leukocytes to ECs and subsequent transmigration of monocytes across the endothelium are considered important steps for the initiation of atherosclerosis. Sun et al. demonstrated that long-term exposure of ApoE−/− mice to low concentrations of PM_2.5_ increased plaque areas and macrophage infiltration [4]. Together, these results not only indicate that fine particles induce the activation of HUVECs and result in monocyte adhesion due to increased expression of adhesion molecules but also imply that fine particles may participate in the development of atherosclerosis. More importantly, our study suggests that Treg cells play a role in attenuating fine particles-mediated vascular inflammation and atherosclerosis.

Fine particles may induce inflammatory responses in human macrophages [36], human epithelial lung cells [37], and human endothelial cells [11, 15]. In this study, increased mRNA and protein expression of IL-6 and IL-8 demonstrates that the fine particles caused inflammatory responses in HUVECs. On the other hand, Treg cells-treated HUVECs showed significantly decreased mRNA and protein expression of IL-6 and IL-8, suggesting that Tregs may protect fine particles-induced inflammatory responses. Based on these results, we conclude that fine particles induced the expression of adhesion molecules and inflammatory cytokines in HUVECs and that these effects were alleviated by treatment with Tregs.

NF-*κ*B signaling is an important pathway that mediates proinflammatory responses [38, 39]. The role of NF-*κ*B in PM-induced inflammatory responses is supported by emerging evidence. Specifically, fine particles derived from diesel engines (diesel exhaust particles) were shown to activate NF-*κ*B in human bronchial epithelium [40–42]. Studies suggested that NF-*κ*B activation induced by diesel exhaust particles is related to the expression of inflammatory chemokines, such as IL-8, monocyte chemoattractant protein-1, and adhesion molecules [43]. In addition, diesel ultrafine particles (UFPs) may also mediate proinflammatory responses via NF-*κ*B activation in endothelial cells [43]. On the contrary, in human antimycobacterial immunity, the NF-*κ*B activity was suppressed by diesel exhaust particles, and consequently antimycobacterial immunity was impaired [44]. Therefore, fine particles may alter the NF-*κ*B activity in a microenvironment-dependent fashion. In our study, after treatment with NF-*κ*B specific inhibitor PDTC, fine particles-induced inflammatory responses were almost completely abolished. Moreover, in agreement with increased expression of adhesion molecules and inflammatory cytokines, the EMSA results also showed that fine particles induced NF-*κ*B activation in HUVECs. In addition, He et al. previously reported that Tregs downregulated ox-LDL/LPS-induced NF-*κ*B activation in HUVECs [18]; similarly, our study demonstrates that Tregs dramatically decreased PM-induced NF-*κ*B activation in HUVECs. Together, these findings imply that Treg cells may decrease fine particles-induced expression of adhesion molecules and inflammatory cytokines mainly by downregulating NF-*κ*B activation.

Some mechanisms about Treg-mediated inhibition that have been found consist of anti-inflammatory cytokines secreted by Treg cells or cell contact-dependent suppression [45]. In our study, TW experiments and neutralizing antibodies were used to explore the mechanisms of Treg-mediated suppression of HUVECs. By blocking physical contact between Tregs and HUVECs (TW), the suppression of inflammatory responses was only partly reversed, indicating that cell contact played a role in Treg-mediated suppression. Moreover, in the supernatants of coculture system, the concentrations of IL-10 and TGF-*β*1 were significantly increased, suggesting that anti-inflammatory cytokines might be required in Treg-mediated suppression. Thus, the reduced NF-*κ*B activation in Treg-treated HUVECs may be partly owing to the increased concentrations of IL-10, because IL-10 could suppress NF-*κ*B activation [46]. After treatment with both anti-IL-10 and TGF-*β*1 mAbs, the suppression of inflammatory responses in TW system was abolished. Therefore, it is speculated that the mechanisms including cell contact and anti-inflammatory cytokines contribute to suppression mediated by Tregs.

In summary, fine particles (SRM2786) may stimulate the expression of adhesion molecules and inflammatory cytokines via NF-*κ*B activation in HUVECs. More importantly, to the best of our knowledge, this present study is the first to demonstrate that Treg cells may protect PM-induced inflammatory responses and downregulate NF-*κ*B activation in HUEVCs via cell contact and anti-inflammatory cytokines* in vitro*. These findings may provide novel targets for treating PM-induced adverse health effects, especially cardiovascular diseases. Future studies are required to investigate the* in vivo* effects of Treg cells on fine particles-induced cardiovascular diseases, such as atherosclerosis, in animal models.

## Figures and Tables

**Table 1:** Primers used for real-time PCR and the size of products.

Genes	Forward (5′-3′)	Reverse (5′-3′)	Size (bp)
VCAM-1	TAAAATGCCTGGGAAGATGG	GGTGCTGCAAGTCAATGAGA	151
ICAM-1	CAGAGGTTGAACCCCACAGT	CCTCTGGCTTCGTCAGAATC	196
IL-6	CAAATTCGGTACATCCTCGACGGC	GGTTCAGGTTGTTTTCTGCCAGTGC	109
IL-8	TAGCAAAATTGAGGCCAAGG	AAACCAAGGCACAGTGGAAC	227
*β*-actin	AGTGTGACGTGGACATCCGC	ACTCGTCATACTCCTGCTTGCTG	243

## Abbreviations

PM: Particulate matter
HUVECs: Human umbilical vein endothelial cells
VCAM-1: Vascular cell adhesion molecule-1
ICAM-1: Intercellular adhesion molecule-1
THP-1: Human acute monocytic leukemia cells
EMSA: Electrophoretic mobility shift assay.
